# A use case study on late stent thrombosis for ontology-based temporal reasoning and analysis

**DOI:** 10.1186/2041-1480-5-49

**Published:** 2014-12-11

**Authors:** Kim Clark, Deepak Sharma, Rui Qin, Christopher G Chute, Cui Tao

**Affiliations:** Boston Scientific Corporation, Maple Grove, MN USA; Division of Biomedical Statistics and Informatics, Mayo Clinic, Rochester, MN USA; School of Biomedical Informatics, University of Texas Health Science Center at Houston, Houston, TX USA

## Abstract

In this paper, we show how we have applied the Clinical Narrative Temporal Relation Ontology (CNTRO) and its associated temporal reasoning system (the CNTRO Timeline Library) to trend temporal information within medical device adverse event report narratives. 238 narratives documenting occurrences of late stent thrombosis adverse events from the Food and Drug Administration’s (FDA) Manufacturing and User Facility Device Experience (MAUDE) database were annotated and evaluated using the CNTRO Timeline Library to identify, order, and calculate the duration of temporal events. The CNTRO Timeline Library had a 95% accuracy in correctly ordering events within the 238 narratives. 41 narratives included an event in which the duration was documented, and the CNTRO Timeline Library had an 80% accuracy in correctly determining these durations. 77 narratives included documentation of a duration between events, and the CNTRO Timeline Library had a 76% accuracy in determining these durations. This paper also includes an example of how this temporal output from the CNTRO ontology can be used to verify recommendations for length of drug administration, and proposes that these same tools could be applied to other medical device adverse event narratives in order to identify currently unknown temporal trends.

## Introduction

The Clinical Narrative Temporal Relation Ontology (CNTRO) [1] and its associated temporal reasoning framework (CNTRO Timeline Library) [2, 3] can be used to facilitate an efficient and semi-automated temporal analysis of events documented within a narrative. Previously it has been shown how CNTRO can be combined with LifeFlow [4] software developed by the University of Maryland, which is capable of visualizing event sequences, such that it is possible to see patterns in the order of events within several narratives [5]. CNTRO’s ability to correctly answer temporal-related questions regarding specific events that have occurred within a narrative has also been previously demonstrated [6]. The goal of this present paper is to illustrate how CNTRO (referring to both the ontology and its associated Timeline Library) can be used to analyze temporal properties of events documented across multiple narratives. In this example, CNTRO is able to verify a recommendation for length of drug administration.

The Food and Drug Administration (FDA) requires notification of all medical device adverse events that are associated with malfunction, serious injury, or death [7]. Events leading up to the device failure are compiled and reported within a narrative text, which is made publically available through the MAUDE (Manufacturer and User Facility Device Experience) database [8, 9]. Analysts at the Center for Devices and Radiological Health (CDRH) read the event histories of each narrative to identify potential trends that may exist, which includes temporal patterns (similar sequences of events, similar durations of or between events, similar time/date stamps of event occurrences, etc.) [10]. However with 80,000 to 120,000 device-related adverse events reported annually to the FDA [11], this approach to trend identification is time consuming, expensive, and the potential exists for a missed trend identification. An automated temporal analysis of adverse event narratives would lead to faster identification of patterns and/or earlier prediction of a future failure, which could be used to drive improvements into the next generation of medical devices.

Automating temporal analysis of events within a narrative is a complex problem. A computer program cannot create a timeline of events and answer time-related questions by querying information directly from a narrative without semantic annotation and inference. Human experts can understand temporal relationships through the use of words such as “before”, “after”, “during”, “following”, etc. and appreciate that 1 year, 12 months, and 365 days are approximately equivalent even though differences in granularity are used. To allow for a “machine-understandable” data representation and exchange of temporal information automatically, the CNTRO System uses a Semantic-Web [12] based framework to apply relationships between events within natural language narratives through the use of the RDF (Resource Description Framework) triple representation [1]. An RDF triple consists of a subject, an object, and a predicate, which indicates the relationship between the subject and the object [10].

Consider the following example. “*60 days after stent implantation, antiplatelet therapy was discontinued in preparation for a splenectomy surgery*.” In this example, stent implantation is identified as the subject, antiplatelet therapy discontinuation is identified as the object, and “after” is identified as the predicate. A temporal relationship is created between stent implantation and discontinuation of antiplatelet therapy using a temporal offset of 60 days.

The computer program now “understands” that stent implantation occurred first, and discontinuation of antiplatelet therapy occurred second. It also “understands” that the time delay between these two events was 60 days. Additionally, there is an inference that because antiplatelet therapy was stopped, it had to have started at some point prior. The CNTRO framework then creates a timeline for events and provides a programmatic query interface to access the timeline information. This makes it possible for the time-related information to now be queried in an automated manner. In our particular example, we could ask questions such as: Which event occurred first? How long after stent implantation was antiplatelet therapy administration discontinued?

Many previous efforts have been attempted to model temporal information within computer-based systems. Ontologies such as Time ontology [13] and the SWRL Temporal ontology [14] can formally model temporal information in general and connect with semantic reasoners for inferring new temporal relations based on semantics defined within the ontologies. These ontologies only focus on structured data with absolute time information, however, and therefore cannot precisely capture the temporal information expressed in human language [1]. In clinical narratives, many temporal features are expressed in relative (e.g. next Friday) or ambiguous (e.g. early last week) ways. Ignoring this data will forgo valuable information that could be otherwise leveraged in clinical research. Models such as the HL7 time specification [15] and the TimeML model [16] offer a way to represent temporal information form semi-structured or unstructured narratives. These approaches, however, do not provide the formal semantic definition capacities for domain knowledge as ontologies do. In clinical narratives, temporal information is often not explicitly expressed, but rather needs to be inferred before the data can be further analyzed. Without a reasoning component, it is difficult to resolve a relatively complete patient history for profound clinical studies [17]. Therefore, we believe that the CNTRO system is necessary as it provides a formal ontology in OWL with well-defined semantics for the time domain and enables semantic-web [12] based temporal reasoning.

## Methods

### The CNTRO system

**Figure 1:**
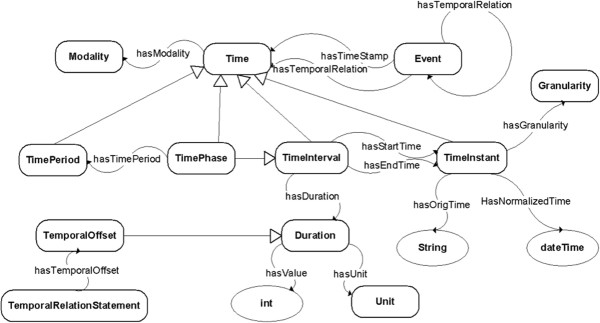
**CNTRO overview.**

CNTRO [1] is an OWL ontology designed to model temporal relations among clinical events. Figure 1 shows the ontology overview. It models clinical events, temporal entities (including time instants, time intervals, repeated time periods, and durations), time granularity (minute, hour, day, month, year), temporal relationships, and time uncertainties in the semantic web notation. In order for users to annotate events and time-related information using CNTRO semantics, a Protégé plug-in, Semantator [18], was developed to interface with CNTRO’s temporal reasoning framework. Semantator provides users two modes: manual annotation mode and semi-automatic annotation mode. In the manual annotation mode, the users can view the domain ontology, the document to be annotated, and the annotated result in the same environment. For semi-automatic annotation, we have linked Semantator with Natural Language Processing tools that support automatic named entity recognition. Users can browse, revise, and save the annotation results at anytime. Semantator has been used to create gold standard annotations for evaluating the reasoning output in our project. The annotated information is stored as an OWL/RDF file or an RDF triple. The CNTRO Timeline Library is then used to infer temporal information not explicitly expressed in the original narrative [2]. The CNTRO Timeline Library contains a rule-based normalizer that automatically converts different temporal expressions into standard formats such as the XML dateTime format. It also leverages the semantic definitions in the ontology (e.g. OWL DL axioms, property transitivity and inversions) to support temporal relation inference. These OWL features were handled by the Pellet OWL reasoner. In addition, the Timeline Library contains a set of Java functions for answering a list of time-related questions, such as when a particular event happened, chronological sequence of events, durations of events, durations between events, temporal relations between events, and sorting a set of events on the timeline. The Timeline Library first calls the reasoner to infer new temporal relations. It then considers all the temporal relations among the events, normalized timestamps of the events, and durations of the events to compute new time stamps of events if possible. It can also calculate the duration of an event given the start and end time of the event and the duration between two events given the time stamps of them. After all the possible inferences and calculations are done, it tries to sort a given set of events based on all the above information.

### Late stent thrombosis adverse event identification

Late Stent Thrombosis (LST) adverse event narratives were used to demonstrate how the CNTRO system and its automated temporal relation reasoning can be used to verify drug therapy duration recommendations. Although the exact mechanism or mechanisms of LST are not known, it has been observed to occur less frequently when dual antiplatelet therapy has been administered over a period of time [19, 20]. Current guidelines recommend the administration of dual antiplatelet therapy for 3 to 6 months following drug-eluting stent implantation, unless the patient is not at high risk for bleeding, in which case therapy is recommended for 12 months [21]. The CNTRO System was used to evaluate the order of events within each narrative and query both the duration in which antiplatelet therapy was administered and the duration between initial stent implantation and the occurrence of late stent thrombosis.

Narratives used in this study were obtained from medical device adverse event reports documented within the MAUDE database. 238 adverse event reports were identified in which late stent thrombosis occurred, defined either as “late” within the report or by a duration of 6 months between stent implantation and the occurrence of thrombosis. These narratives were then manually annotated using Semantator by an expert.

### Adverse event narrative annotation

We created a domain ontology which includes common events that occur after stent implantation was created with specific normalized event types. The domain ontology is relative to simple comparing to the CNTRO. It only defines the set of events we what to monitor for our use case. These events were then imported into CNTRO for temporal relationship modeling. The following events were included: initial stent implantation, follow up stent implantation(s), start and stop time points of antiplatelet therapy administration, unrelated surgeries occurring after stent implantation, late stent thrombosis, myocardial infarction, admission to the emergency room, and patient death. Events such as guide wire insertion are required for all stenting procedures; therefore annotation of these events would not be beneficial and were therefore not performed. Life-saving events following the thrombosis detection were also not annotated within the narratives as the focus of the application of CNTRO was based on verifying the recommended duration of drug administration and not the potential to survive following an occurrence of thrombosis.

Start and stop point of the antiplatelet therapy were annotated to determine the duration of therapy. Unrelated surgeries which occurred between stent implantation and identification of thrombosis, myocardial infarction, admission to the emergency room and patient death were all annotated as events to verify the Event Order and Inferred Relationship functions of the CNTRO Timeline Library.

**Figure 2:**
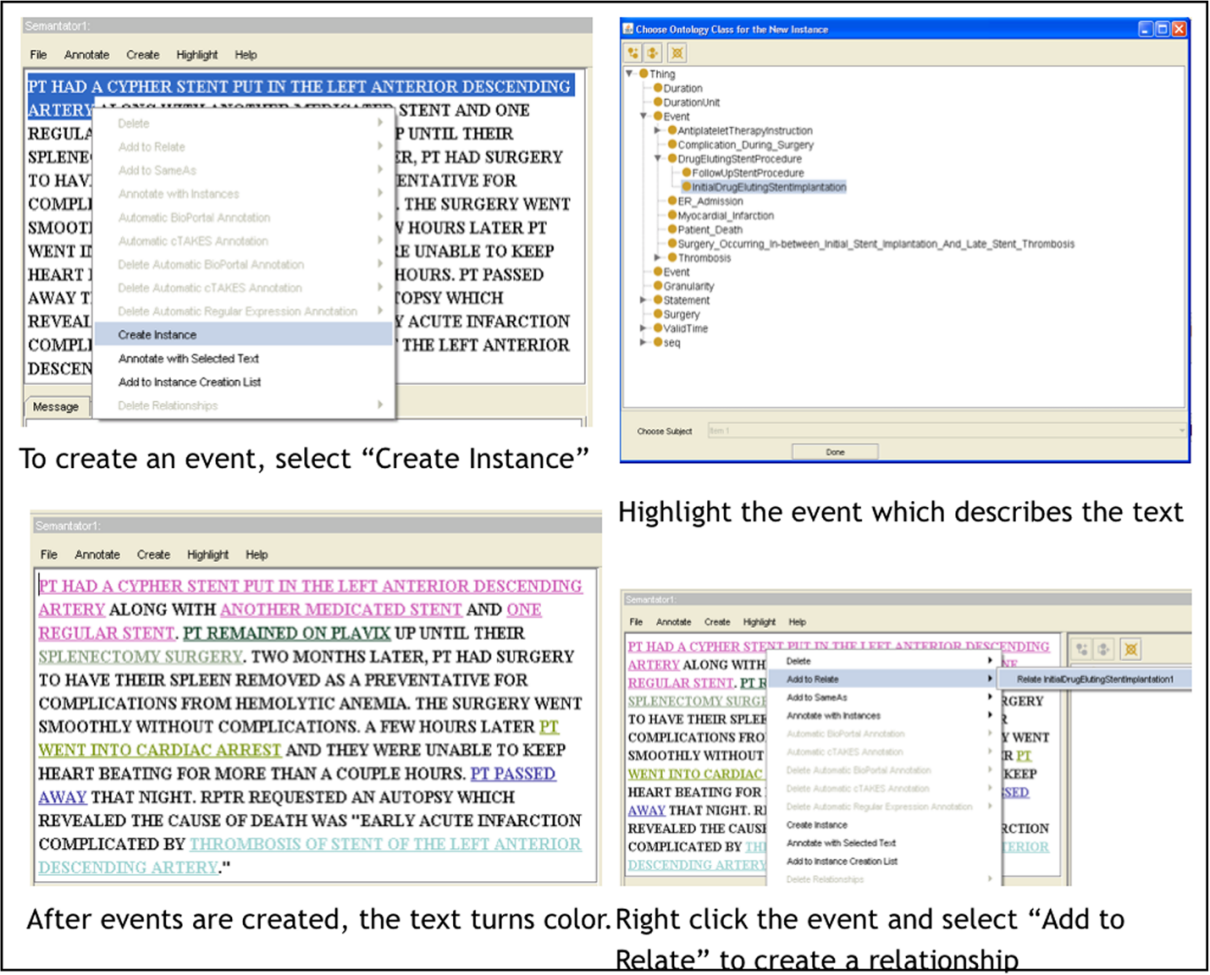
**Annotating an event.**

Annotations were performed using Semantator. The first step in the annotation process involves identification of the individual events. As shown in Figure 2, after each event is created, the text turns color specific to each event type.

**Figure 3:**
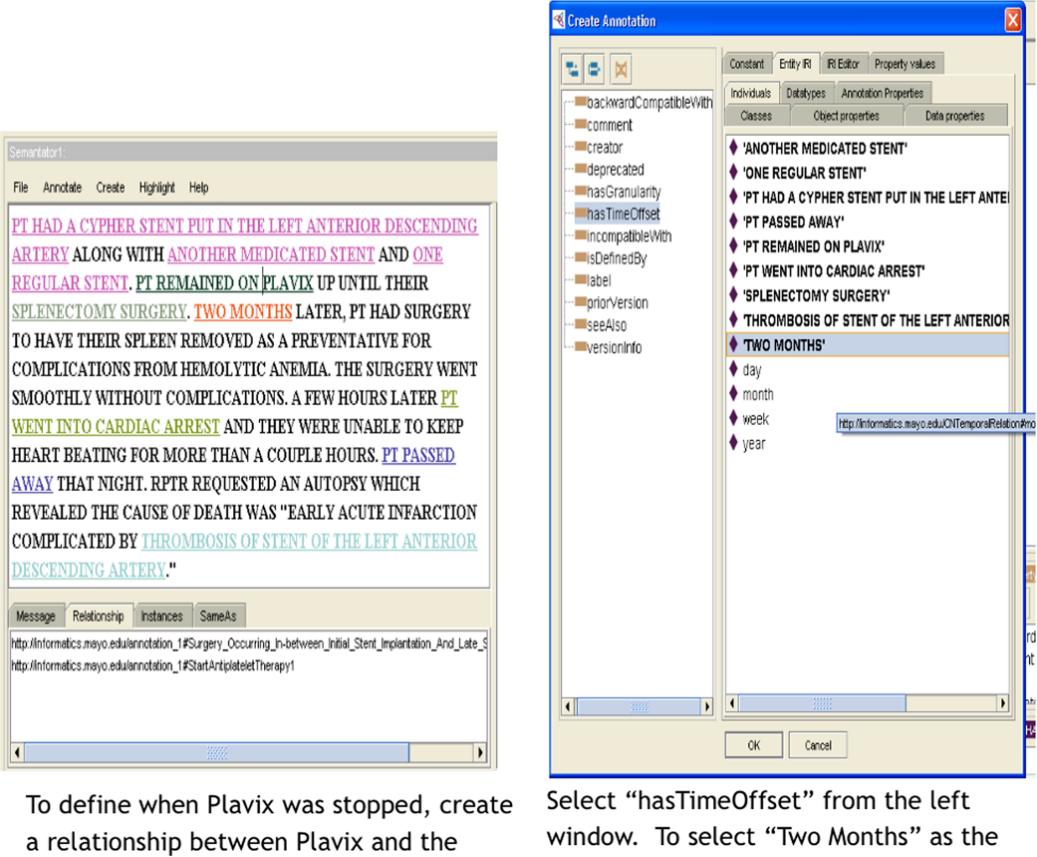
**Annotating a relationship.**

After the events are created, temporal relationships between the events can be defined through annotation, see Figure 3. A relationship connects two events and can indicate that the events occurred or began at the same time, or that one event occurred or began before another event. If the duration between the events is known, this is annotated after the relationship has been defined, see Figure 3. If a specific event has a duration, this information is annotated as well.

### CNTRO timeline evaluation

**Figure 4:**
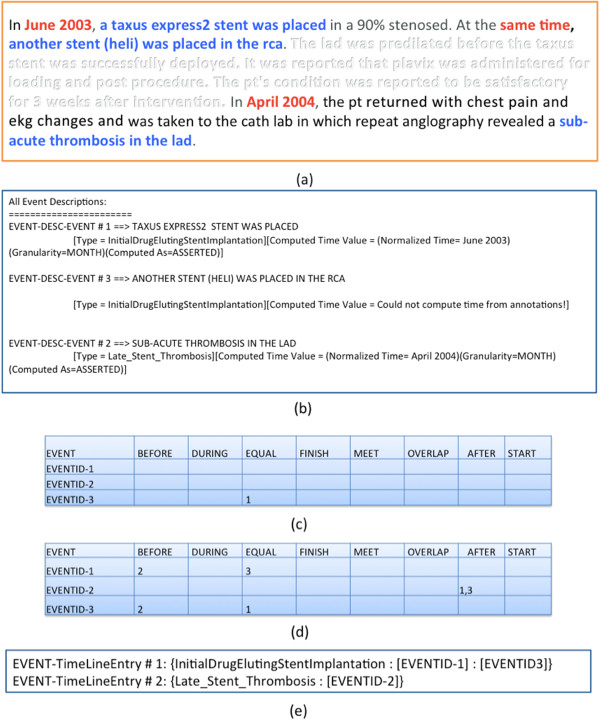
**Sample evaluation matrix (a) original document (partial); (b) event description; (c) asserted temporal relations; (d) asserted and inferred temporal relations; (e) timeline bucket.**

For each annotated narrative, the CNTRO Timeline Library creates a matrix that visually shows the temporal relationships between the events, which is a simple way to track, view, document, and evaluate the accuracy of CNTRO system timeline computations. Each annotated event is included within the matrix. The matrix indicates which events occur at the same time, and then orders the remaining events on a timeline as applicable. Figure 4 shows a sample matrix. Figure 4(a) shows a partial complaint file with three events and their corresponding temporal constraints highlighted. Figure 4(b) shows the event descriptions. Figure 4(c) shows the annotated (asserted) temporal relations between events (e.g., EVENTID-3 EQUAL EVENTID-1). Figure 4(d) shows both asserted and inferred temporal relations between events. Based on the annotation result, the reasoner knows the timestamp of EVENTID-1 and EVENTID-3 as well as the fact that EVENTID-3 and EVENTID-1 happened at the same time. It can therefore infer the timestamp of EVENTID-3 which is the same as the one for EVENTID-1. Then based on the timestamps, it can infer the temporal relations among the three events. Finally Figure 4(e) shows the timeline bucket that includes a set of sorted timeline entries.

The annotations of the Late Stent Thrombosis Adverse Event Narratives were reviewed using these matrices and compared against gold standard results, in which events were manually recorded in timeline order from two exports reading each narrative. The timeline accuracy was assessed by comparing the gold standard results to the CNTRO Timeline Library results. All conflicting results between CNTRO and the gold standard were reviewed among the human experts to determine if the conflict resulted from an error in the gold standard result, an error in manually annotations, or an error in the reasoning component of CNTRO.

### CNTRO duration evaluation

Durations can be computed for an individual event, between two events, or between an event and a timestamp. CNTRO first determines if ‘start’ and ‘end’ time information exists for an event to calculate the duration. If one of these pieces of information is missing, the program then computes it by either using a duration annotation, “*Antiplatelet therapy was administered for two months*” (the antiplatelet therapy event is defined here with a duration of 2 months) or uses a temporal relation to another event with a relative time stamp, “*Antiplatelet therapy was started in May 2006. In July 2006, the patient underwent prostrate surgery. Antiplatelet therapy was stopped the day before surgery”.* In this second example the occurrence of antiplatelet therapy starting and stopping each have a time stamp, and CNTRO infers that antiplatelet therapy was administered for 2 months based on the duration between the start and end times. In some cases, the duration of a pair of events cannot be calculated directly (the two events are not directly connected through the RDF graph), but need to go through one or more intermediate events. In this case, the above two functions need to be called iteratively until the duration of the two events are calculated.

The adverse event narratives for late stent thrombosis could describe durations in days, months, and/or years. Month was the most frequent granularity used in the complaint data, followed by years, and then days. To be able to compare data from different narratives, the duration granularity was normalized to ‘Month’ for this use case as this was the most frequently used granularity, and estimating durations reported in years by number of days would likely increase the noise within the data. The durations calculated by CNTRO were compared to manual calculations to determine accuracy.

### Application of CNTRO temporal analysis

To provide an example of how the CNTRO system can potentially be used to evaluate temporal properties within narrative data, survival analysis was performed using the narratives that specified both a duration of antiplatelet therapy and time from stent implantation to late stent thrombosis (or in which a duration could be inferred) to examine therapeutic guidelines for antiplatelet administration duration. Note that as this data comes from the FDA MAUDE Database, all records within the example ended up with an event of late stent thrombosis. Data of patients who have not had a late stent thrombosis occurrence are not easily accessible; therefore this example is purely illustrative of the CNTRO system’s capability. Similarly, because the data used within this analysis comes from adverse event files indicating thrombosis occurred, no patient data requires censoring.

Late Stent Thrombosis adverse event files were divided into two different groups based on how long antiplatelet therapy was administered in patients following implantation of a drug-eluting stent. Using current antiplatelet therapy recommendations, any adverse event narrative specifying that antiplatelet medication was administered for less than 6 months was segregated into the Shorter Duration of Antiplatelet Therapy group. Any adverse event narrative indicating that antiplatelet medication was administered for 6 or more months was segregated into the Longer Duration of Antiplatelet Therapy group. Adverse event narratives that did not provide information specifying how long antiplatelet therapy was prescribed were excluded from the analysis.

## Results

### CNTRO timeline and duration evaluation

238 adverse event narratives included at least two events, such that a timeline could be created within CNTRO for system evaluation. For each narrative, the CNTRO system-inferred timeline was evaluated with a gold standard result. The CNTRO system was capable of correctly ordering each event in all but 8 of the narratives. This resulted in an overall CNTRO timeline accuracy of 95%. There were 41 adverse event narratives that included enough information such that the duration of antiplatelet therapy was known. The CNTRO automatic reasoning system had an 80% accuracy in inferring and/or calculating this duration of an event. There were 77 adverse event narratives that included enough information such that the duration between stent implantation and identification of late stent thrombosis was known. The CNTRO Automatic reasoning system had a 76% accuracy in inferring and/or calculating this duration between events. An evaluation of the errors and discussion of possible enhancements to the CNTRO system is included within the Discussion section.

### Late stent thrombosis adverse event temporal pattern analysis

Within this paper, the CNTRO system was used to confirm what has been previously identified as a temporal pattern within the late stent thrombosis adverse event in a semi-automated manner, which is more efficient than through manual observation. The common event pattern within late stent thrombosis adverse events (stent implantation, administration of antiplatelet therapy, discontinuation of antiplatelet therapy, late stent thrombosis) was shown by CNTRO system through timeline identification of events. This result shows that the CNTRO system has the potential to be applied across multiple adverse event failure modes to identify new trends that have previously not been observed.

There were 36 adverse events that specified both the duration between drug-eluting stent implantation and occurrence of late stent thrombosis, and the duration of antiplatelet therapy. These 36 reports were used to execute a survival analysis. Although this represents only a limited subset of late stent thrombosis events and does not include patient information for those who have not had late stent thrombosis, the data can still be used for illustration purposes of CNTRO’s temporal analysis capabilities. Late Stent Thrombosis adverse event files were divided into two different groups based on how long antiplatelet therapy was administered in patients with an implanted drug-eluting stent. Adverse event narratives that did not provide information specifying how long antiplatelet therapy was prescribed were excluded from the analysis. 14 adverse events reported that antiplatelet therapy was administered for 6 months or less following initial stent implantation. 22 adverse events reported that antiplatelet therapy was administered greater than 6 months.

**Figure 5:**
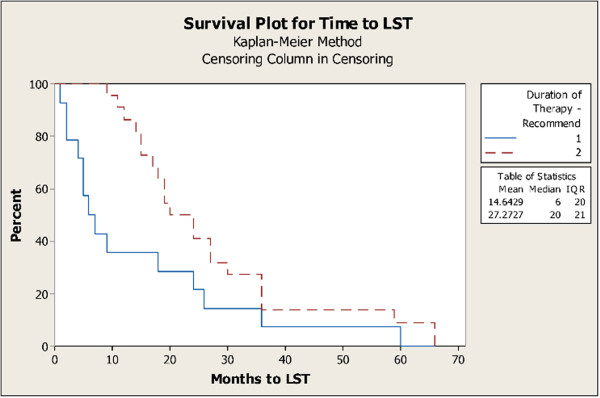
**Survival analysis of shorter duration of antiplatelet therapy (group 1) and longer duration of antiplatelet therapy (group 2) in late stent thrombosis adverse events.**

Survival analysis with Kaplan-Meier curve and log-rank test was performed in Minitab. The median time to LST is 27.3 months for longer antiplatelet therapy group and 14.6 months for shorter antiplatelet therapy group, respectively. The p-value of log-rank test is 0.029, which indicates a significant association between duration of antiplatelet therapy and time to LST. Figure 5 supports that on average, late stent thrombosis occurs later in patients who continued to take antiplatelet therapy longer than 6 months. Although this is a retrospective observational study of a subset of LST cases only, the finding is consistent and supports guidance for use of longer antiplatelet therapy [1]. This example validates the CNTRO System’s ability to confirm known temporal trends and verify drug administration duration recommendations.

## Discussion

### Error analysis

Although the CNTRO system can provide relatively good results for our use case, there are still limitations in the system. First, the evaluation results work well with the MAUDE reports because these reports are relatively short and simple compared to other clinical narratives such as clinical notes. Second, since the purpose of this study is to evaluate CNTRO’s representation and reasoning capacities, the reports were annotated manually. Many ambiguities and uncertainties were resolved during the annotation process. Nevertheless, this study provides promising results and valuable analysis for us to continue develop the CNTRO system.

The CNTRO system was able to order the event sequences for 95% of the narratives. The reasoner failed due to different interpretations of time intervals and background assumptions in the manual annotation. Computing the order of two events is difficult when using ‘start’ or ‘finish’ temporal relations when both the start and end times cannot be annotated. For example, a narrative might specify that antiplatelet therapy began at the time of stent implantation, and specify that it occurred for a period of 2 months. The temporal relation of the event1 (antiplatelet therapy) and event2 (stent implantation) depends on whether the start and end times of the events can be compared. When considering the start time, the two events start at the same time (event1 starts event2). The system cannot infer the relationship by the end time since the duration of “stent implantation” is not specified, given that it occurs at a single point in time. Given the assumption that the stent implantation procedure cannot last for 2 months, we can infer that event1 ends after event2. This kind of background knowledge needs to be further specified in the domain ontology so that the CNTRO system can infer the correct order. Additionally, “patient death” inherently is known to be the last event in a patient-care timeline. This kind of inherited order needs to be incorporated in the domain ontology so that the sequence of events can be correctly inferred.

For duration inference, there are three major reasons the program failed to return the correct results. (1) Annotation ambiguities: some narratives contain duration information in an ambiguous way such as in range (e.g., 2-3 month), or in different levels of granularity (e.g., “two month and ten days”) that the program cannot automatically process. We are working on expanding the ontology so that it can cover ranges. In addition, we are adding more functions to the reasoner so it can normalize durations in different levels of granularity. (2) Long series of events: sometimes the duration calculation involves a long series of events. The program sometimes fails when there are many intermediate events between the start and the end events. This is usually due to one or more intermediate events were not annotated by the ontology and therefore were not included during the reasoning process. 3) Temporal relation granularity: an annotator can specify the level of granularity over a temporal relation. For example, we can specify that the granularity of “event1 before event2” is “day”. This means that the temporal relation was compared on the granularity of day, which implies that although event1 was before event2, but they happened on the same day. This assumption was not programmed in the CNTRO reasoning system yet, and caused errors when calculating the duration between event1 and event3. For example, we know that Event3 may have occurred 183 days after event2, but without the assumption that event1 and event2 happened on the same day, the system cannot infer the duration between event1 and event3. The CNTRO reasoner needs to be updated to handle level of granularity on temporal relations.

### Areas for improvement of MAUDE database for temporal analysis

There were some weaknesses identified regarding the use of adverse event narratives from the MAUDE database. The MAUDE database does not have selectable fields for Device Manufacturer or Brand Name. Due to the free text fields, there are a variety of spellings and misspellings for both the Device Manufacturer and Brand Name which may have resulted in a missed late stent thrombotic adverse event based on how these fields were used to sort complaints. The level of detail in some adverse event narratives was very limited and the duration between stent implantation and stent thrombosis may not have been documented. Additionally, due to patient privacy some time stamps were removed making the duration between stent implantation and stent thrombosis unknown. It is possible that late stent thrombosis occurred in some patients but the complaint narratives were filtered out due to not being able to classify the event as “late.” Late stent thrombosis adverse events may also have been missed while filtering from the files if a different term was used within the narrative as there is no searchable failure mode within MAUDE specific to thrombosis.

### Future CNTRO applications

Of interest in recent literature is a current investigation into understanding whether there is a link between incomplete stent apposition (ISA) (separation between the stent strut and the vessel wall) and late stent thrombosis. Stent which are not adequately apposed following implantation are referred to as acute ISA, and may be due to incorrect stent sizing or inadequate expansion of the stent. Inadequate stent apposition identified at a later point in time is referred to as late ISA. Late ISA can either be persistent, meaning that it was the result of inadequate stent expansion, or acquired, meaning the vessel becomes enlarged, or plaque or thrombosis in-between the stent and wall dislodged creating space, or the stent recoiled. There will likely be future studies attempting to link late stent thrombosis with either persistent or acquired ISA. The CNTRO system could be of value in this investigation to determine if there is a correlation of post-dilation frequency with late stent thrombosis or a relationship between the change in apposition and the duration between discontinuation of antiplatelet therapy and thrombus formation.

## Conclusion

Although the CNTRO system was able to provide relatively good results for this use case, there are still limitations in the system. First, the evaluation results work well with the MAUDE reports because these reports are relatively short and simple compared to other narratives such as clinical notes. More CNTRO system evaluation needs to be performed using complex electronic health record data. Second, since the purpose of this study is to evaluate CNTRO’s representation and reasoning capacities, the reports were annotated manually. The current manual annotation method is not practical for long-term use, and an automatic annotation process is currently under development. Third, many ambiguities were resolved during the annotation process. Uncertainty reasoning is currently being incorporated into the CNTRO system to resolve these ambiguities. In spite of these limitations, this study provides promising results and valuable analysis to support continuing the development of the CNTRO system.
